# Off-Target Influences of Arch-Mediated Axon Terminal Inhibition on Network Activity and Behavior

**DOI:** 10.3389/fncir.2020.00010

**Published:** 2020-03-25

**Authors:** Christopher K. Lafferty, Jonathan P. Britt

**Affiliations:** ^1^Department of Psychology, McGill University, Montreal, QC, Canada; ^2^Center for Studies in Behavioral Neurobiology, Concordia University, Montreal, QC, Canada

**Keywords:** optogenetics, ArchT, nucleus accumbens, photoinhibition, reward-seeking

## Abstract

Archaerhodopsin (ArchT)-mediated photoinhibition of axon terminals is commonly used to test the involvement of specific long-range neural projections in behavior. Although sustained activation of this opsin in axon terminals has the unintended consequence of enhancing spontaneous vesicle release, it is unclear whether this desynchronized signaling is consequential for ArchT’s behavioral effects. Here, we compare axon terminal and cell body photoinhibition of nucleus accumbens (NAc) afferents to test the utility of these approaches for uncovering pathway-specific contributions of neural circuits to behavior. First, in brain slice recordings we confirmed that ArchT photoinhibition of glutamatergic axons reduces evoked synaptic currents and increases spontaneous transmitter release. A further consequence was increased interneuron activity, which served to broadly suppress glutamate input *via* presynaptic GABA_B_ receptors. *In vivo*, axon terminal photoinhibition increased feeding and reward-seeking behavior irrespective of the afferent pathway targeted. These behavioral effects are comparable to those obtained with broad inhibition of NAc neurons. In contrast, cell body inhibition of excitatory NAc afferents revealed a pathway-specific contribution of thalamic input to feeding behavior and amygdala input to reward-seeking under extinction conditions. These findings underscore the off-target behavioral consequences of ArchT-mediated axon terminal inhibition while highlighting cell body inhibition as a valuable alternative for pathway-specific optogenetic silencing.

## Introduction

The nucleus accumbens (NAc) is a forebrain structure that regulates the vigor of reward-seeking. Its excitatory inputs likely encode motivational states and the presence of reward-associated cues (Mannella et al., 2013). For example, paraventricular thalamic (PVT) input regulates food-seeking behavior under conditions of hunger and threat (Labouèbe et al., 2016; Choi and McNally, 2017; Do-Monte et al., 2017; Cheng et al., 2018; Choi et al., 2019), while basolateral amygdala (BLA) input encodes the motivational value of reward-associated cues (Ambroggi et al., 2008; Stuber et al., 2011; Esber and Holland, 2014). Few studies, however, have directly compared the behavioral consequences of pathway-specific manipulations, so it remains unclear how each input distinctly contributes to effective reward-seeking. Maladaptive alterations in the strength of NAc inputs are thought to underlie discrete aspects of psychopathologies, including the aversive symptoms of drug withdrawal (Neumann et al., 2016; Zhu et al., 2016) and stress susceptibility in animal models of depression (Bagot et al., 2015). Thus, pathway-specific inactivation of these inputs is critical to gaining insight into how this circuitry contributes to healthy and unhealthy behavior alike.

Archaerhodopsin (ArchT)-mediated photoinhibition of axon terminals is commonly used to test the involvement of specific long-range neural projections in behavior. Sustained activation of this outward proton pump in axon terminals effectively decreases evoked transmitter release but alkalizes affected axon terminals, which has the unintended consequence of increasing spontaneous vesicle release (El-Gaby et al., 2016; Mahn et al., 2016). It is unclear whether ArchT’s off-target effects undermine the interpretation of its behavioral effects or still permit assessment of pathway-specific function. If the aberrant spontaneous vesicle release recruits local circuit feedforward inhibition, as previously suggested (Mahn et al., 2016), the intended pathway-specific nature of the manipulation may be compromised.

The shortcomings of ArchT terminal inhibition have been well characterized in acute slice preparations (El-Gaby et al., 2016; Mahn et al., 2016), but *in vivo* applications of this technique are still widely used to study the circuit-level basis of specific behaviors (Herrera et al., 2016; Yamamoto and Tonegawa, 2017; Mangieri et al., 2018), particularly concerning NAc inputs (Stefanik et al., 2016; Zhu et al., 2016; Reed et al., 2018; Trouche et al., 2019). Thus, there remains a need to validate the pathway-specific nature of this manipulation in behaving animals.

Here, we compare photoinhibition targeted to the axon terminals or cell bodies of NAc inputs. We test the efficacy of these two approaches for uncovering pathway-specific contributions of the PVT-NAc and BLA-NAc pathways to behavior. We first demonstrate in brain slice recordings that ArchT photoinhibition of glutamatergic fibers effectively reduced evoked excitatory synaptic currents. We also report that it increased asynchronous transmitter release and consequently interneuron spiking, which broadly suppressed glutamate release *via* presynaptic GABA_B_ receptors. *In vivo*, excitatory axon terminal photoinhibition increased feeding and effortful reward-seeking irrespective of the pathway targeted. These effects are comparable to those obtained with broad inhibition (O’Connor et al., 2015) or lesions (Bowman and Brown, 1998) of NAc projection neurons. In contrast, cell body inhibition of NAc afferents from the PVT and BLA revealed pathway-specific contributions to distinct aspects of reward-seeking when food was available and during extinction, respectively. These data underscore the off-target behavioral consequences of ArchT-mediated terminal inhibition while highlighting cell body inhibition as a valuable alternative for pathway-specific optogenetic silencing.

## Materials and Methods

### Experimental Model and Subject Details

Adult wild-type and transgenic C57BL/6J mice were used, including tdTomato Cre-reporter mice (JAX#007914) and parvalbumin-Cre mice (JAX#008069, Jackson Laboratory, Sacramento, CA, USA). All animals were bred in-house and kept on a reverse light cycle with a 12 h photoperiod. Animals underwent surgery at approximately 3 months of age (25–30 g). Six weeks later they were placed on a restricted feeding schedule and maintained at 85–90% of their pre-surgery body weight. The number of male and female mice were balanced within groups. All experiments were conducted following the Canadian Council of Animal Care and the McGill Animal Care Committee.

### Method Details

#### Viral Constructs and Surgery

Before surgery, animals were anesthetized using a ketamine (Ventoquinol, 100 mg/kg) and xylazine (Bayer, 10 mg/kg) cocktail. The skull of the animal was then secured to a stereotaxic frame (Kopf Instruments) and prepared for intracranial virus injections according to the standard stereotaxic procedure. Seven-hundred nanoliter of virus (5.0 × 10^12^ GC/ml) was injected bilaterally over 10 min using a Nanoject II Injector with an oil-filled glass micropipette pulled to a tip diameter of 10 μm (Drummond Scientific, 3-000-203-G/X).

For axonal photoinhibition experiments, rAAV5-CaMKIIα-eArchT3.0-eYFP (UNC Vector Core) was delivered into the BLA (AP −1.8 mm, ML ±2.8 mm, DV −5.15 mm) and PVT (AP −1.1 mm, ML ±0.35 mm, DV −3.3 mm) of different cohorts of wildtype mice. Optical fibers with a 200 μm core were implanted in the NAc 10 min later (10° angle, AP 1.5 mm, ML ±1.35 mm, DV −4.6 mm). Animals used for brain slice electrophysiology experiments included PV reporter mice and were prepared in the same manner, but optical fibers were not implanted.

For afferent-specific cell body photoinhibition experiments, retroAAV2-CAG-ArchT-GFP (Neurophotonics Centre at Université Laval) was delivered to the NAc (AP 1.5 mm, ML ±0.62 mm, DV −4.7 to −4.2) and an optical fiber was placed above the BLA (AP −2.06 mm, ML ±3 mm, DV −4.02 mm) or PVT (10° angle, AP −1.20 or −0.95 mm, ML ±0.56 mm, DV −2.82 mm) of different cohorts of wildtype mice.

#### Behavioral Testing

Mice were trained in sound-attenuating chambers (Med Associates), in which levers were extended on either side of a centrally located food receptacle. A house light and speaker were located on the opposite side of the chamber. All behavioral data were collected using the Med Associates software.

Food-restricted mice were tethered to optical fiber and placed in these operant chambers daily for 40-min sessions. One lever was randomly designated the active lever. Initially, each press on this lever was reinforced with the delivery of 30 μl of a 15% sucrose solution (m/v) to the food receptacle and a tone presentation (4.8 kHz, 80 dB, 5 s duration). After mice earned 40 rewards in a single session, we switched them to a variable ratio (VR3) reinforcement schedule. The number of active lever presses required for reward delivery and tone presentation then varied randomly between 1 and 5. Inactive lever presses were always inconsequential. Both levers remained extended throughout each session.

Photoinhibition experiments were carried out after animals consistently attained 20 rewards per daily session. Photoinhibition involved bilateral intracranial light delivery (532 nm, 10 mW) for two 8-min periods within the 40-min session. The timing of the photoinhibition periods was counterbalanced across 2 days of testing. Animals subsequently experienced two sessions under extinction conditions, in which presses on the active lever were no longer reinforced. Both extinction sessions were preceded by three daily sessions on the VR3 reinforcement schedule.

#### Brain Slice Electrophysiology

Mice were anesthetized and perfused with a modified artificial cerebrospinal fluid that contained, in mM, 92 NMDG, 20 HEPES, 25 glucose, 30 NaHCO_3_, 1.25 NaH_2_PO_4_, 2.5 KCL, 5 sodium ascorbate, 3 sodium pyruvate, 2 thiourea, 10 MgSO_4_, 0.5 CaCl_2_ (pH 7.35). Two-hundred micrometer thick coronal slices containing the NAc were prepared using a VT-1200 vibratome (Leica) and held at 34°C for 10 min in this same solution. Slices were then transferred to a “holding ACSF” at room temperature, which was identical except that NaCl (92 mM) was included instead of NMDG and the MgSO_4_ and CaCl_2_ concentrations were 1 and 2 mM, respectively. The ACSF used on the patch rig was maintained at 31°C and contained, in mM, 119 NaCl, 2.5 KCL, 1.25 NaH_2_PO_4_, 2 MgSO_4_, 2 CaCl_2_, 24 NaHCO_3_, and 12.5 glucose. All ACSF preparations were saturated with 95% O_2_ and 5% CO_2_. Cells were visualized on an upright microscope with infrared differential interference contrast and fluorescence microscopy. Whole-cell patch-clamp recordings were made using a MultiClamp 700B amplifier using 2 kHz lowpass Bessel filter and 10 kHz digitization with pClamp 11 software (Molecular Devices). Recordings were made using glass pipets with resistance 4.0–6.0 MΩ, filled with an internal solution containing, in mM, 117 cesium methanesulfonate, 20 HEPES, 0.4 EGTA, 2.8 NaCl, 5 TEA-Cl, 4 Mg-ATP and 0.4 Na-GTP (pH 7.3). Projection neurons identified by morphology, membrane resistance, and hyperpolarized resting membrane potential were patched in the NAc shell in areas with bright eYFP fluorescence. Patched cells were held at −70 mV to record evoked and spontaneous EPSCs or at 0 mV to record spontaneous IPSCs. sEPSCs were recorded over 10 min (5 min per condition). sIPSCs were recorded over 30 min. EPSCs were evoked in pairs (50 ms interval) once per minute for 30 min with a single stimulating electrode positioned 100–200 mm dorsal to the recorded neuron. Recordings that included photoinhibition and CGP55984 lasted 15 min (5 min per condition). Series resistance (10–25 MΩ) remained stable throughout data collection. To record interneuron spiking, cell-attached recordings were carried out on tdTomato^+^ neurons of PV-reporter mice in the NAc shell in areas with bright eYFP fluorescence. Recordings of spiking activity took place over 10 min (5 min per condition). Opsin activation was achieved with 590 nm light (2 mw, ThorLabs, DC4104) directed through the microscope objective.

#### Histology

At the end of each behavioral experiment, animals were anesthetized with 270 mg/kg Euthansol (Merck) and transcardially perfused with 4% paraformaldehyde (PFA, Sigma-Aldrich). Brains were removed, post-fixed in PFA for 24 h, and then transferred to PBS for 48 h. Tissue was then sliced into 60 μm sections on a vibratome (Leica VT1000s) and mounted on microscope slides with a MOWIOL plus DAPI (Sigma-Adrich, St. Louis, MO, USA) solution.

### Quantification and Statistical Analysis

For electrophysiological recordings, all data were normalized to the last 3 min of the baseline condition of a given recording. The frequency of sEPSCs, sIPSCs, and spiking was calculated by counting the number of events that occurred in 1-min bins across each recording. Once normalized, these data were used for all time-course graphs. For summary graphs, the mean normalized frequency was calculated for each experimental condition.

Two-tailed paired *t*-tests and two-way ANOVAs were used for statistical comparisons of behavior across photoinhibition conditions and across pathways.

Sidak’s multiple comparisons tests were conducted for all ANOVA *post hoc* tests. The significance of all statistical tests was determined using *α* = 0.05. All data are reported as the mean ± SEM.

## Results

### ArchT-Mediated Axonal Inhibition of NAc Afferents Increases Reward-Seeking Behavior

**Figure 1:**
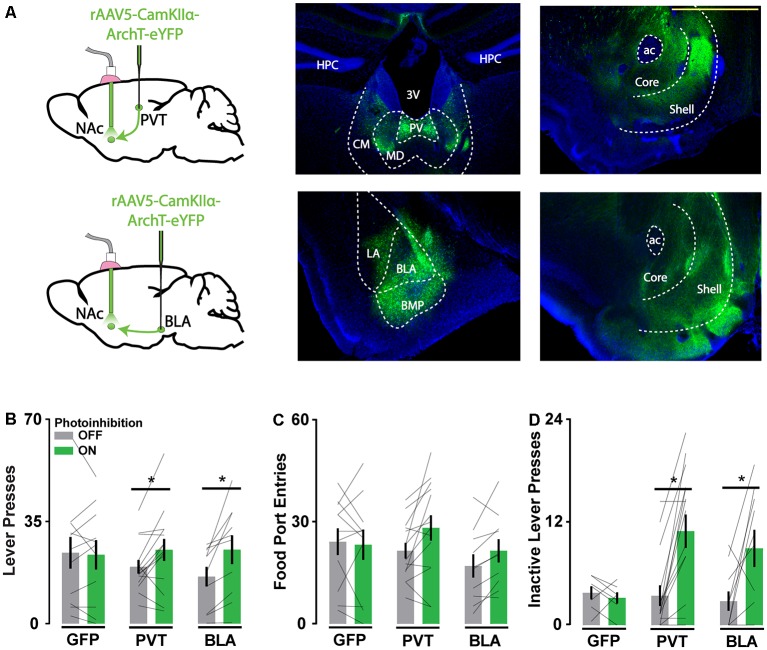
Archaerhodopsin (ArchT)-mediated axon terminal inhibition of glutamate afferents in the Nucleus accumbens (NAc) increases reward-seeking behavior. **(A)** Schematic of viral injections and optic probe placements (*left*). Representative coronal brain slices showing ArchT-eYFP expression in PVT and BLA neurons (*middle*) and their associated axons in the NAc (right). Scale bar, 500 μm. **(B,C)** Photoinhibition of PVT and BLA axons increased active lever responses (*n*_GFP_ = 11; *n*_PVT_ = 14; *n*_BLA_ = 10; *F*_(2,32)_ = 3.96, *p* < 0.05; for significant *post hoc* tests *t*_(32)_ > 2.63) but not food port entries when reward was available (*F*_(2,32)_ = 2.52, *p* = 0.10). **(D)** During extinction, photoinhibition of PVT and BLA axons increased inactive lever responses (*n*_GFP_ = 7; *n*_PVT_ = 15; *n*_BLA_ = 10; *F*_(2,29)_ = 4.70, *p* < 0.05; for significant *post hoc* tests *t*_(29)_ > 3.31). Error bars represent SEM. *Signifies *p* < 0.05. 3V, third ventricle; ac, anterior commissure; BLA, basolateral amygdala; BMP, posterior basomedial amygdaloid nucleus; CM, central medial thalamic nucleus; HPC, hippocampus; LA, lateral amygdaloid nucleus; MD, mediodorsal thalamic nucleus; PV, paraventricular thalamic nucleus.

The PVT-NAc pathway is thought to integrate hunger signals (Kelley et al., 2005; Kirouac, 2015; Labouèbe et al., 2016; Meffre et al., 2019) while the BLA-NAc pathway processes reward-predictive stimuli (Ambroggi et al., 2008; Esber and Holland, 2014; Beyeler et al., 2018), so we hypothesized that inhibition of these pathways would differentially influence the vigor of food-seeking and responsivity to reward-associated cues, respectively. We used an axon terminal photoinhibition strategy to test this idea, bilaterally targeting light to the NAc of mice expressing ArchT in their PVT or BLA (Figure 1A). We trained the mice to lever press for food reward on a variable ratio schedule of reinforcement (VR3) and compared their lever press and food port responses within behavioral sessions, across periods with and without intracranial light delivery. We also assessed the behavioral impact of photoinhibition during interspersed extinction sessions when lever presses were not reinforced.

Photoinhibition of PVT and BLA axons in the NAc increased the frequency of active lever pressing (Figures 1B,C), whereas intracranial light delivery in GFP-only control mice did not affect reward-seeking behavior. Photoinhibition of PVT and BLA axons also increased inactive lever responding during extinction sessions (Figure 1D), consistent with general disinhibition of behavior. These behavioral effects are comparable to those obtained with direct NAc neuron photoinhibition (O’Connor et al., 2015) and NAc lesions (Bowman and Brown, 1998), which suggests that any inhibitory influence on NAc physiology may similarly disinhibit reward-seeking. Alternatively, given the known off-target effects of ArchT-mediated axonal inhibition, this approach to inhibiting specific pathways may generate broad, unintended disturbances in NAc physiology.

### Axon Terminal Inhibition of Excitatory NAc Afferents Increases Local Inhibitory Signaling

**Figure 2:**
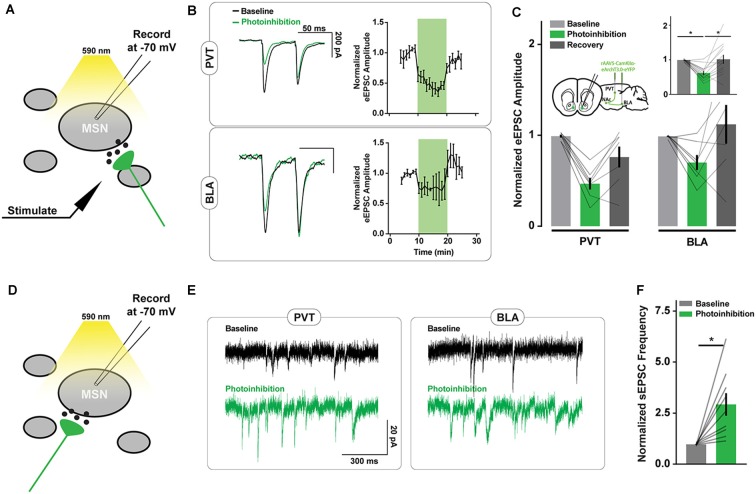
ArchT-mediated inhibition of excitatory axons in the NAc increases spontaneous excitatory postsynaptic currents (sEPSC) frequency. **(A)** Schematic of brain slice recording conditions where electrically-evoked-EPSCs were recorded from NAc spiny neurons before, during, and after Arch-mediated photoinhibition of excitatory axon terminals. **(B)** Example recordings from NAc neurons showing changes in the amplitude of electrically-evoked EPSCs during photoinhibition of PVT (*top left*) and BLA (*bottom left*) axons in the NAc. Summary of relative change in EPSC amplitudes (*right*) over time in response to photoinhibition during minutes 10–20. **(C)** Summary of effect of photoinhibition on EPSC amplitudes. Inset shows data collapsed across pathways, highlighting main effect of photoinhibition [*n*_PVT_ = 7(3 animals); *n*_BLA_ = 7(3); *F*_(2,24)_ = 10.40, *p* < 0.001; t_baseline vs. inhibition (24)_ = 4.18*; t_baseline vs. inhibition (24)_ = 3.68*]. **(D)** Schematic of brain slice recording conditions where spontaneous EPSCs were recorded from NAc spiny neurons during the Arch-mediated photoinhibition of excitatory axon terminals. **(E)** Example NAc neuron recordings highlighting the increase in spontaneous EPSCs that accompanied Arch-mediated inhibition of PVT and BLA axons. **(F)** Summary of effect of photoinhibition on normalized sEPSC frequency [*n* = 9(3); *t*_(8)_ = 3.48, *p* < 0.0]. Error bars represent SEM. *Signifies *p* < 0.01. MSN, the medium spiny neuron.

We examined the off-target consequences of the ArchT photoinhibition of PVT and BLA axons in NAc neuron brain slice recordings (Figure 2A). We first confirmed that opsin activation in PVT and BLA axons reduced the amplitude of electrically-evoked excitatory postsynaptic currents (eEPSCs) in NAc neurons (Figures 2B,C), consistent with the intended purpose of the manipulation. We next monitored the frequency of spontaneous EPSCs (sEPSCs) and found it to be elevated during periods of ArchT activation (Figures 2D–F), consistent with previous results (Mahn et al., 2016). There was no change in the amplitude or decay time of spontaneous synaptic currents in response to photoinhibition (Supplementary Figures S1A–D), nor was there any effect of light on eEPSCs or sEPSCs in recordings from wildtype animals (Supplementary Figures S2A,B).

**Figure 3:**
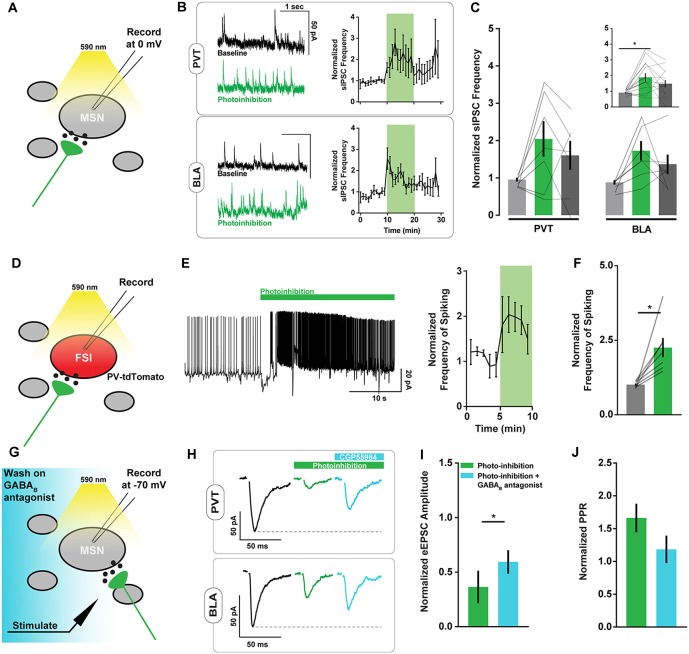
ArchT-mediated inhibition of excitatory axons in the NAc increases spiny neuron spontaneous inhibitory postsynaptic currents (sIPSC) frequency and PV+ interneuron spiking. **(A)** Schematic of brain slice recording conditions where spontaneous IPSCs were recorded from NAc spiny neurons before, during, and after Arch-mediated photoinhibition of excitatory axon terminals. **(B)** Example NAc neuron recordings showing changes in sIPSC frequency during photoinhibition of PVT (*top* *left*) and BLA (*bottom left*) axons in the NAc. Summary of relative change in sIPSC frequency over time in response to photoinhibition (*right*). **(C)** Summary of effect of photoinhibition on sIPSC frequency. Inset shows data collapsed across pathways, highlighting main effect of photoinhibition [*n*_PVT_ = 7(3 animals); *n*_BLA_ = 7(3); *F*_(2,24)_ = 7.26, *p* < 0.01; t_baseline vs. inhibition (24)_ = 3.79*]. **(D)** Schematic of brain slice recording conditions where spiking activity was recorded in tdTomato-labeled PV+ fast-spiking interneurons (FSIs) during Arch-mediated photoinhibition of excitatory afferent inputs. **(E)** Example recording from a PV+ interneuron in the NAc showing elevated spiking activity coincident with Arch-mediated axon terminal photoinhibition (*left*). Summary of relative change in FSI spiking frequency over time in response to photoinhibition (*right*). **(F)** Summary of effect of photoinhibition on normalized interneuron spiking [*n* = 7(2); *t*_(6)_ = 3.64, *p* < 0.05]. **(G)** Schematic of brain slice recording conditions, where electrically-evoked EPSCs were recorded from NAc spiny neurons during Arch-mediated photoinhibition of excitatory axon terminals in the presence of a GABA_B_ antagonist (CGP55984). **(H)** Example NAc neuron recordings showing the effects of a GABA_B_ antagonist on evoked EPSC amplitudes during photoinhibition of PVT (*top*) and BLA axons (*bottom*). **(I,J)** Summary of effect of GABA_B_ antagonist on evoked EPSC amplitude [*n* = 3(2); *t*_(2)_ = 5.51, *p* < 0.05] and normalized pulse-paired ratio (PPR; *t*_(2)_ = 2.90, *p* = 0.10) during photoinhibition of excitatory axon terminals. Baseline data not shown. Error bars represent SEM. *Signifies *p* < 0.05. FSI, fast-spiking interneuron; MSN, the medium spiny neuron.

Possibly the asynchronous glutamate release may not directly alter the firing rate of NAc projection neurons, since they have a resting membrane potential close to −80 mV and are thought to fire action potentials upon concerted excitatory input (Goto and Grace, 2008). However, certain interneuron populations in the NAc are highly excitable and may be responsive to small changes in excitatory signaling. To evaluate whether interneuron activity is affected by ArchT-mediated increases in asynchronous glutamate release, we monitored spontaneous inhibitory postsynaptic currents (sIPSCs) in NAc neurons. The frequency but not the amplitude of sIPSCs was elevated during PVT and BLA axon photoinhibition (Figures 3A–C, Supplementary Figure S1B), consistent with the hypothesis that local inhibitory signaling is upregulated by unintended glutamate release from ArchT-expressing fibers.

To more directly evaluate this hypothesis, we recorded the spiking activity of parvalbumin-positive (PV) interneurons in the NAc of PV-tdTomato mice in response to ArchT photoinhibition of PVT and BLA axons (Figure 3D). We found a sharp increase in PV interneuron spiking following opsin activation, irrespective of its localization to PVT or BLA axons (Figures 3E,F). While it is unclear if this increase in spiking is a direct consequence of asynchronous glutamate release, it suggests that activation of ArchT in any collection of excitatory axons in the NAc may cause common disruptions in NAc physiology.

While excess PV interneuron activity directly inhibits NAc projection neurons, it may also broadly suppress glutamate release *via* presynaptic GABA_B_ receptors located on excitatory afferents (Kupferschmidt and Lovinger, 2015). Indeed, bath application of the GABA_B_ receptor antagonist CGP55984 at 2 μM partially reversed the effects of axonal ArchT activation on eEPSC amplitude and pulse paired ratios (Figures 3G–J) without affecting baseline measures of eEPSC amplitude or sEPSC frequency (Supplementary Figure S3). This result raises further doubt about the pathway-specificity of the axonal inhibition approach.

### Cell Body Inhibition of NAc Afferents Reveals Pathway-Specific Contributions of PVT and BLA Inputs to Reward-Seeking

**Figure 4:**
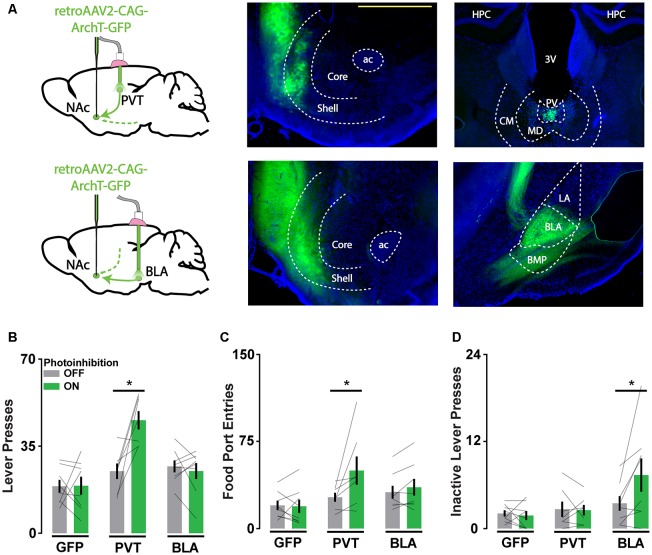
Cell body inhibition of NAc afferents reveals pathway-specific contributions of PVT and BLA inputs to reward-seeking. **(A)** Schematic of viral injections and optic probe placements (*left*). Representative coronal brain slices showing ArchT-GFP expression in axon terminals in the NAc (*middle*) and in soma that project to the NAc in the PVT and BLA (*right*). Scale bar, 500 μm. **(B,C)** Photoinhibition of NAc-projecting PVT neurons increases active lever responses (*n*_GFP_ = 8; *n*_PVT_ = 7; *n*_BLA_ = 8; *F*_(2,20)_ = 8.31, *p* < 0.01, *t*_PVT__(20)_ = 4.68*) and food port entries (*F*_(2,20)_ = 3.76, *p* < 0.05; *t*_PVT__(20)_ = 3.47*). **(D)** During extinction, photoinhibition of NAc-projecting BLA neurons increases inactive lever responses (*F*_(2,20)_ = 4.32, *p* < 0.05; *t*_BLA__(20)_ = 3.44*). Error bars represent SEM. *Signifies *p* < 0.05. 3V, third ventricle; ac, anterior commissure; BLA, basolateral amygdala; BMP, posterior basomedial amygdaloid nucleus; CM, central medial thalamic nucleus; HPC, hippocampus; LA, lateral amygdaloid nucleus; MD, mediodorsal thalamic nucleus; PV, paraventricular thalamic nucleus.

To re-evaluate the validity of the behavioral findings we obtained with axonal ArchT activation, we repeated the experiments described above using an alternative approach to disrupting pathway function. Capitalizing on the efficient axon terminal-infecting virus retroAAV, we drove ArchT expression in all NAc-projecting neurons and targeted light to the PVT or BLA (Figure 4A). This method provided pathway specificity, but the manipulation occurred upstream of the NAc. These animals were trained and tested in the same manner as before, and we evaluated the differential influence of PVT and BLA pathways on operant reward-seeking behavior.

With light directed to the PVT and BLA in different cohorts of mice, we found that photoinhibition of the PVT-NAc pathway but not the BLA-NAc pathway increased lever press and food port responding when food was available (Figures 4B,C). This result was consistent with previous findings that implicate PVT input in the integration of hunger signals that regulate the vigor of food-seeking. In contrast, photoinhibition of the BLA-NAc pathway but not the PVT-NAc pathway increased inactive lever responding during extinction when food was not available (Figure 4D). This finding is consistent with the role of BLA input in regulating cue-reward associations. Together, these results suggest that soma-targeted photoinhibition is a valuable alternative to axon-targeted photoinhibition for assessing pathway-specific contributions to behavior, as only the former revealed dissociable influences of PVT and BLA afferents to the NAc on reward-seeking.

## Discussion

In trying to identify dissociable influences of PVT and BLA input to the NAc on reward-seeking behavior, we compared axon-targeted and soma-targeted photoinhibition strategies using the outward proton pump ArchT. We found that axon terminal inhibition increased reward-seeking behavior similarly when targeted to either pathway, whereas pathway-specific inhibition of upstream cell bodies produced dissociable behavioral effects that were consistent with previous literature. NAc neuron brain slice recordings confirmed that activation of ArchT in excitatory axon terminals reduces eEPSCs yet increases sEPSCs. The pathway-specific nature of this manipulation was undermined by a concomitant increase in GABAergic interneuron activity, which was associated with a broad GABA_B_ receptor-dependent reduction in eEPSC amplitudes. These results suggest that the ArchT photoinhibition of excitatory axons has off-target consequences that generally disrupt NAc physiology, which may explain why similar changes in behavior result from the inhibition of distinct axonal inputs. Soma-targeted photoinhibition appears to be a valuable alternative for pathway-specific optogenetic silencing.

The NAc integrates excitatory input from several limbic structures (Mannella et al., 2013) and is a convergent site of dysregulation in many psychiatric disorders (Ahmari et al., 2013; Bagot et al., 2015; Francis et al., 2015; Creed et al., 2016; Neumann et al., 2016). Identifying the distinct behavioral contributions of these excitatory inputs is challenging because they similarly engage NAc physiology (Britt et al., 2012) and originate in regions that are themselves highly interconnected (Pitkänen et al., 2000; Li and Kirouac, 2008; Do-Monte et al., 2015). The study of these circuit elements thus heavily relies on our ability to selectively disrupt pathway-specific function. Unfortunately, many of the optogenetic tools commonly used to silence long-range neural projections have unclear limitations *in vivo*, and their off-target effects may mask pathway-specific differences, particularly in highly integrative structures such as the NAc. Accordingly, there is considerable value in comparing different pathway-specific inhibition strategies for manipulating multiple, parallel long-range projections.

### Alternatives to Targeting ArchT Photoinhibition to Axon Terminals

Optogenetic approaches for silencing neural activity have repeatedly been found to produce off-target effects and unpredictable changes in network activity that preclude straightforward interpretations of experimental outcomes. For example, tissue heating in response to prolonged light delivery is sufficient to alter potassium conductance in striatal neurons (Owen et al., 2019). Additionally, ion transporters such as ArchT and halorhodopsin can dramatically alter the intracellular ionic environment affecting the pH level and chloride reversal potential, respectively (Raimondo et al., 2012; Mahn et al., 2016). These disruptions are most pronounced in axons on account of their relatively small intracellular volume. A separate issue is the rebound excitation that often follows any acute hyperpolarization (Arrenberg et al., 2009).

Fortunately, new tools and experimental approaches have mitigated some of these unintended effects. The development of highly effective retrograde viral vectors (Tervo et al., 2016) has facilitated projection-specific cell body photoinhibition strategies, as demonstrated here. This approach benefits from the capacity of the soma to buffer against significant changes in pH and ionic composition (Wiegert et al., 2017). Opsin expression can be further restricted with intersectional strategies involving recombinase proteins such as Cre or FLP and retrograde viral vectors. This approach may be necessary when targeting specific pathways within reciprocally connected brain regions.

Anion-conducting channelrhodopsins (ACRs) may be the best option currently available for photoinhibition experiments. Since their conductance is dependent on the membrane potential, their activation can shunt voltage fluctuations of the cell without inducing strong hyperpolarization or significantly altering the ionic environment (Berndt et al., 2016). The light-driven chloride channels that have been engineered (eACRs; Berndt et al., 2014) or found in nature (GtACRs; Govorunova et al., 2015) are also more light-sensitive than any presently used ion transporter opsins. Unfortunately, chloride channels appear to be excitatory in many axons, due to locally elevated chloride concentrations (Khirug et al., 2008; Malyshev et al., 2017). Soma-restricted GtACR variants have been developed to circumvent this issue, but their expression level has to be optimized to minimize their excitatory influence in the axon hillock (Mahn et al., 2018).

These new tools have fewer drawbacks than their predecessors, but it remains difficult to interpret the effects of inhibiting specific projections that are embedded in recurrent circuitry (Spellman et al., 2015; Do-Monte et al., 2017). It is unclear how silencing a pathway will ultimately affect downstream neurons, let alone the network as a whole. Limiting the duration of photoinhibition may mitigate some concerns, but the organization of the affected circuitry and behavioral state of the animal should be carefully considered.

### PVT and BLA Inputs to the NAc Influence Discrete Aspects of Reward-Seeking

PVT and BLA projections to the NAc have been found to promote and discourage reward-seeking in different contexts (Stuber et al., 2011; Millan et al., 2015; Zhu et al., 2016; Do-Monte et al., 2017; Bercovici et al., 2018; Reed et al., 2018; Shen et al., 2019). For instance, photostimulation of PVT input to the NAc can reduce or increase sucrose-seeking behavior (Labouèbe et al., 2016; Do-Monte et al., 2017; Cheng et al., 2018), while photostimulation of different fibers in the BLA-NAc pathway can generate place preference or aversion (Shen et al., 2019). These mixed effects likely reflect the heterogeneity of the PVT and BLA. By studying coarse disruptions of pathway activity alongside manipulations of genetically and spatially defined subpopulations in varied contexts (Labouèbe et al., 2016; Shen et al., 2019), we can begin to understand the net contributions of these pathways to behavior.

Here, we identified a unique role of PVT inputs in modulating the vigor of food-seeking behavior, which is consistent with a wealth of literature implicating this structure in integrating hunger signals and regulating feeding (Kelley et al., 2005; Stratford and Wirtshafter, 2013; Kirouac, 2015; Labouèbe et al., 2016; Choi and McNally, 2017; Do-Monte et al., 2017; Cheng et al., 2018; Meffre et al., 2019). While stimulation of a smaller glucose-sensing subset of PVT-NAc projectors can promote consumptive behavior (Labouèbe et al., 2016), we find that the net effect of bulk PVT-NAc inhibition is an increase in food-seeking, consistent with the net reductions in PVT-NAc pathway activity that have been observed in the rostral NAc during feeding (Reed et al., 2018). Gross excitatory drive from this input may, therefore, gate food-seeking behaviors by downregulating NAc activity overall, while smaller, genetically defined populations—like those that sense low levels of interstitial glucose (Labouèbe et al., 2016)—may target relatively circumscribed areas of the NAc which have opposing influences on feeding behavior.

Our soma-targeted photoinhibition of the BLA-NAc pathway did not affect lever-pressing behavior when food was available but increased it under extinction conditions, consistent with the role of this pathway in regulating operant responding following changes in outcome (Shiflett and Balleine, 2010). BLA-NAc activity may thus have a pronounced role in behavioral suppression under varied conditions. Overall, the use of increasingly refined tools to inhibit projection-specific activity will aid efforts to dissect neural circuit function concerning behavior.

## Data Availability Statement

The datasets generated for this study are available on request to the corresponding author.

## Ethics Statement

The animal study was reviewed and approved by Canadian Council of Animal Care and the McGill Animal Care Committee.

## Author Contributions

CL and JB conceived the study, designed the experiments, and wrote the manuscript. CL conducted the experiments. JB supervised the research.

## Conflict of Interest

The authors declare that the research was conducted in the absence of any commercial or financial relationships that could be construed as a potential conflict of interest.

## Supplementary Material

FIGURE S1 ArchT-mediated photoinhibition of excitatory axons in the NAc does not affect the amplitude or the decay rate of spontaneous synaptic currents. Related to Figures 2, 3. Summary of effect of ArchT axon terminal inhibition on **(A)** the amplitude of spontaneous EPSCs [*n* = 9(3 animals); *t*_(8)_ = 0.13, *p* = 0.90], **(B)** the decay rate of spontaneous EPSCs (*n* = 8(3); *t*_(7)_ = 1.61, *p* = 0.15], **(C)** the amplitude of spontaneous IPSCs [*n* = 14(6); *t*_(13)_ = 1.22, *p* = 0.25], and **(D)** the decay rate of spontaneous IPSCs [*n* = 14(6); *t*_(13)_ = 0.46, *p* = 0.65].

FIGURE S2 Light alone does not affect the amplitude of eEPSCs or the frequency of sEPSCs in NAc cell recordings from wildtype animals. Related to Figure 2. Summary of the effect of light on **(A)** the amplitude of evoked EPSCs [*n* = 8(3 animals); *t*_(7)_ = 2.11, *p* = 0.07] and **(B)** the frequency of spontaneous EPSCs (*t*_(7)_ = 0.63, *p* = 0.55).

FIGURE S3 In the absence of photoinhibition, a GABAB antagonist does not affect the amplitude of eEPSCs or the frequency of sEPSCs recorded from NAc neurons of wildtype animals. Related to Figure 3. Summary of the effect of light on **(A)** the amplitude of evoked EPSCs [*n* = 5(3 animals); *t*_(4)_ = 0.80, *p* = 0.47] and **(B)** the frequency of spontaneous EPSCs (*t*_(4)_ = 0.08, *p* = 0.94).

## References

[B1] Ahmari S. E. Spellman T. Douglass N. L. Kheirbek M. A. Simpson H. B. Deisseroth K.. (2013). Repeated cortico-striatal stimulation generates persistent OCD-like behavior. Science 340, 1234–1239. 10.1126/science.1234733 23744948 PMC3954809

[B2] Ambroggi F. Ishikawa A. Fields H. L. Nicola S. M. (2008). Basolateral amygdala neurons facilitate reward-seeking behavior by exciting nucleus accumbens neurons. Neuron 59, 648–661. 10.1016/j.neuron.2008.07.004 18760700 PMC2603341

[B3] Arrenberg A. B. Del Bene F. Baier H. (2009). Optical control of zebrafish behavior with halorhodopsin. Proc. Natl. Acad. Sci. U S A 106, 17968–17973. 10.1073/pnas.0906252106 19805086 PMC2764931

[B4] Bagot R. C. Parise E. M. Pena C. J. Zhang H. X. Maze I. Chaudhury D.. (2015). Ventral hippocampal afferents to the nucleus accumbens regulate susceptibility to depression. Nat. Commun. 6:7062. 10.1038/ncomms8062 25952660 PMC4430111

[B5] Bercovici D. A. Princz-Lebel O. Tse M. T. Moorman D. E. Floresco S. B. (2018). Optogenetic dissection of temporal dynamics of amygdala-striatal interplay during risk/reward decision making. eNeuro 5:ENEURO.0422-18.2018. 10.1523/ENEURO.0422-18.2018 30627636 PMC6325538

[B6] Berndt A. Lee S. Y. Ramakrishnan C. Deisseroth K. (2014). Structure-guided transformation of channelrhodopsin into a light-activated chloride channel. Science 344, 420–424. 10.1126/science.1252367 24763591 PMC4096039

[B7] Berndt A. Lee S. Y. Wietek J. Ramakrishnan C. Steinberg E. E. Rashid A. J.. (2016). Structural foundations of optogenetics: determinants of channelrhodopsin ion selectivity. Proc. Natl. Acad. Sci. U S A 113, 822–829. 10.1073/pnas.1523341113 26699459 PMC4743797

[B8] Beyeler A. Chang C. J. Silvestre M. Leveque C. Namburi P. Wildes C. P.. (2018). Organization of valence-encoding and projection-defined neurons in the basolateral amygdala. Cell Rep. 22, 905–918. 10.1016/j.celrep.2017.12.097 29386133 PMC5891824

[B9] Bowman E. M. Brown V. J. (1998). Effects of excitotoxic lesions of the rat ventral striatum on the perception of reward cost. Exp. Brain Res. 123, 439–448. 10.1007/s002210050588 9870603

[B10] Britt J. P. Benaliouad F. McDevitt R. A. Stuber G. D. Wise R. A. Bonci A. (2012). Synaptic and behavioral profile of multiple glutamatergic inputs to the nucleus accumbens. Neuron 76, 790–803. 10.1016/j.neuron.2012.09.040 23177963 PMC3607383

[B11] Cheng J. Wang J. Ma X. Ullah R. Shen Y. Zhou Y. D. (2018). Anterior paraventricular thalamus to nucleus accumbens projection is involved in feeding behavior in a novel environment. Front. Mol. Neurosci. 11:202. 10.3389/fnmol.2018.00202 29930498 PMC5999750

[B13] Choi E. A. Jean-Richard-Dit-Bressel P. Clifford C. W. G. Mcnally G. P. (2019). Paraventricular thalamus controls behavior during motivational conflict. J. Neurosci. 39, 4945–4958. 10.1523/JNEUROSCI.2480-18.2019 30979815 PMC6670259

[B12] Choi E. A. McNally G. P. (2017). Paraventricular thalamus balances danger and reward. J. Neurosci. 37, 3018–3029. 10.1523/JNEUROSCI.3320-16.2017 28193686 PMC6596734

[B14] Creed M. Ntamati N. R. Chandra R. Lobo M. K. Luscher C. (2016). Convergence of reinforcing and anhedonic cocaine effects in the ventral pallidum. Neuron 92, 214–226. 10.1016/j.neuron.2016.09.001 27667004 PMC8480039

[B15] Do-Monte F. H. Minier-Toribio A. Quinones-Laracuente K. Medina-Colon E. M. Quirk G. J. (2017). Thalamic regulation of sucrose seeking during unexpected reward omission. Neuron 94, 388.e4–400.e4. 10.1016/j.neuron.2017.03.036 28426970 PMC5484638

[B16] Do-Monte F. H. Quiñones-Laracuente K. Quirk G. J. (2015). A temporal shift in the circuits mediating retrieval of fear memory. Nature 519, 460–463. 10.1038/nature14030 25600268 PMC4376623

[B17] El-Gaby M. Zhang Y. Wolf K. Schwiening C. J. Paulsen O. Shipton O. A. (2016). Archaerhodopsin selectively and reversibly silences synaptic transmission through altered pH. Cell Rep. 16, 2259–2268. 10.1016/j.celrep.2016.07.057 27524609 PMC4999416

[B18] Esber G. R. Holland P. C. (2014). The basolateral amygdala is necessary for negative prediction errors to enhance cue salience, but not to produce conditioned inhibition. Eur. J. Neurosci. 40, 3328–3337. 10.1111/ejn.12695 25135841

[B19] Francis T. C. Chandra R. Friend D. M. Finkel E. Dayrit G. Miranda J.. (2015). Nucleus accumbens medium spiny neuron subtypes mediate depression-related outcomes to social defeat stress. Biol. Psychiatry 77, 212–222. 10.1016/j.biopsych.2014.07.021 25173629 PMC5534173

[B20] Goto Y. Grace A. A. (2008). Limbic and cortical information processing in the nucleus accumbens. Trends Neurosci. 31, 552–558. 10.1016/j.tins.2008.08.002 18786735 PMC2884964

[B21] Govorunova E. G. Sineshchekov O. A. Janz R. Liu X. Spudich J. L. (2015). Natural light-gated anion channels: a family of microbial rhodopsins for advanced optogenetics. Science 349, 647–650. 10.1126/science.aaa7484 26113638 PMC4764398

[B22] Herrera C. G. Cadavieco M. C. Jego S. Ponomarenko A. Korotkova T. Adamantidis A. (2016). Hypothalamic feedforward inhibition of thalamocortical network controls arousal and consciousness. Nat. Neurosci. 19, 290–298. 10.1038/nn.4209 26691833 PMC5818272

[B23] Kelley A. E. Baldo B. A. Pratt W. E. (2005). A proposed hypothalamic-thalamic-striatal axis for the integration of energy balance, arousal and food reward. J. Comp. Neurol. 493, 72–85. 10.1002/cne.20769 16255002

[B24] Khirug S. Yamada J. Afzalov R. Voipio J. Khiroug L. Kaila K. (2008). GABAergic depolarization of the axon initial segment in cortical principal neurons is caused by the Na-K-2Cl cotransporter NKCC1. J. Neurosci. 28, 4635–4639. 10.1523/JNEUROSCI.0908-08.2008 18448640 PMC6670448

[B25] Kirouac G. J. (2015). Placing the paraventricular nucleus of the thalamus within the brain circuits that control behavior. Neurosci. Biobehav. Rev. 56, 315–329. 10.1016/j.neubiorev.2015.08.005 26255593

[B26] Kupferschmidt D. A. Lovinger D. M. (2015). Inhibition of presynaptic calcium transients in cortical inputs to the dorsolateral striatum by metabotropic GABA_B_ and mGlu2/3 receptors. J. Physiol. 593, 2295–2310. 10.1113/JP270045 25781000 PMC4457193

[B27] Labouèbe G. Boutrel B. Tarussio D. Thorens B. (2016). Glucose-responsive neurons of the paraventricular thalamus control sucrose-seeking behavior. Nat. Neurosci. 19, 999–1002. 10.1038/nn.4331 27322418 PMC4964931

[B28] Li S. Kirouac G. J. (2008). Projections from the paraventricular nucleus of the thalamus to the forebrain, with special emphasis on the extended amygdala. J. Comp. Neurol. 506, 263–287. 10.1002/cne.21502 18022956

[B29] Mahn M. Gibor L. Patil P. Cohen-Kashi Malina K. Oring S. Printz Y.. (2018). High-efficiency optogenetic silencing with soma-targeted anion-conducting channelrhodopsins. Nat. Commun. 9:4125. 10.1038/s41467-018-06511-8 30297821 PMC6175909

[B30] Mahn M. Prigge M. Ron S. Levy R. Yizhar O. (2016). Biophysical constraints of optogenetic inhibition at presynaptic terminals. Nat. Neurosci. 19, 554–556. 10.1038/nn.4266 26950004 PMC4926958

[B31] Malyshev A. Y. Roshchin M. V. Smirnova G. R. Dolgikh D. A. Balaban P. M. Ostrovsky M. A. (2017). Chloride conducting light activated channel GtACR2 can produce both cessation of firing and generation of action potentials in cortical neurons in response to light. Neurosci. Lett. 640, 76–80. 10.1016/j.neulet.2017.01.026 28093304

[B32] Mangieri L. R. Lu Y. Xu Y. Cassidy R. M. Xu Y. Arenkiel B. R.. (2018). A neural basis for antagonistic control of feeding and compulsive behaviors. Nat. Commun. 9:52. 10.1038/s41467-017-02534-9 29302029 PMC5754347

[B33] Mannella F. Gurney K. Baldassarre G. (2013). The nucleus accumbens as a nexus between values and goals in goal-directed behavior: a review and a new hypothesis. Front. Behav. Neurosci. 7:135. 10.3389/fnbeh.2013.00135 24167476 PMC3805952

[B34] Meffre J. Sicre M. Diarra M. Marchessaux F. Paleressompoulle D. Ambroggi F. (2019). Orexin in the posterior paraventricular thalamus mediates hunger-related signals in the nucleus accumbens core. Curr. Biol. 29, 3298.e4–3306.e4. 10.1016/j.cub.2019.07.069 31543448

[B35] Millan E. Z. Reese R. M. Grossman C. D. Chaudhri N. Janak P. H. (2015). Nucleus accumbens and posterior amygdala mediate cue-triggered alcohol seeking and suppress behavior during the omission of alcohol-predictive cues. Neuropsychopharmacology 40, 2555–2565. 10.1038/npp.2015.102 25872917 PMC4569945

[B36] Neumann P. A. Wang Y. Yan Y. Wang Y. Ishikawa M. Cui R.. (2016). Cocaine-induced synaptic alterations in thalamus to nucleus accumbens projection. Neuropsychopharmacology 41, 2399–2410. 10.1038/npp.2016.52 27074816 PMC4946070

[B37] O’Connor E. C. Kremer Y. Lefort S. Harada M. Pascoli V. Rohner C.. (2015). Accumbal D1R neurons projecting to lateral hypothalamus authorize feeding. Neuron 88, 553–564. 10.1016/j.neuron.2015.09.038 26593092

[B38] Owen S. F. Liu M. H. Kreitzer A. C. (2019). Thermal constraints on *in vivo* optogenetic manipulations. Nat. Neurosci. 22, 1061–1065. 10.1038/s41593-019-0422-3 31209378 PMC6592769

[B39] Pitkänen A. Pikkarainen M. Nurminen N. Ylinen A. (2000). Reciprocal connections between the amygdala and the hippocampal formation, perirhinal cortex and postrhinal cortex in rat. A review. Ann. N Y Acad. Sci. 911, 369–391. 10.1111/j.1749-6632.2000.tb06738.x 10911886

[B40] Raimondo J. V. Kay L. Ellender T. J. Akerman C. J. (2012). Optogenetic silencing strategies differ in their effects on inhibitory synaptic transmission. Nat. Neurosci. 15, 1102–1104. 10.1038/nn.3143 22729174 PMC3428858

[B41] Reed S. J. Lafferty C. K. Mendoza J. A. Yang A. K. Davidson T. J. Grosenick L.. (2018). Coordinated reductions in excitatory input to the nucleus accumbens underlie food consumption. Neuron 99, 1260.e4–1273.e4. 10.1016/j.neuron.2018.07.051 30146308

[B42] Shen C. J. Zheng D. Li K. X. Yang J. M. Pan H. Q. Yu X. D.. (2019). Cannabinoid CB_1_ receptors in the amygdalar cholecystokinin glutamatergic afferents to nucleus accumbens modulate depressive-like behavior. Nat. Med. 25:350. 10.1038/s41591-019-0372-z 30700866

[B43] Shiflett M. W. Balleine B. W. (2010). At the limbic-motor interface: disconnection of basolateral amygdala from nucleus accumbens core and shell reveals dissociable components of incentive motivation. Eur. J. Neurosci. 32, 1735–1743. 10.1111/j.1460-9568.2010.07439.x 21044174 PMC2994582

[B44] Spellman T. Rigotti M. Ahmari S. E. Fusi S. Gogos J. A. Gordon J. A. (2015). Hippocampal-prefrontal input supports spatial encoding in working memory. Nature 522, 309–314. 10.1038/nature14445 26053122 PMC4505751

[B45] Stefanik M. T. Kupchik Y. M. Kalivas P. W. (2016). Optogenetic inhibition of cortical afferents in the nucleus accumbens simultaneously prevents cue-induced transient synaptic potentiation and cocaine-seeking behavior. Brain Struct. Funct. 221, 1681–1689. 10.1007/s00429-015-0997-8 25663648 PMC5259736

[B46] Stratford T. R. Wirtshafter D. (2013). Injections of muscimol into the paraventricular thalamic nucleus, but not mediodorsal thalamic nuclei, induce feeding in rats. Brain Res. 1490, 128–133. 10.1016/j.brainres.2012.10.043 23111346 PMC3529785

[B47] Stuber G. D. Sparta D. R. Stamatakis A. M. Van Leeuwen W. A. Hardjoprajitno J. E. Cho S.. (2011). Excitatory transmission from the amygdala to nucleus accumbens facilitates reward seeking. Nature 475, 377–380. 10.1038/nature10194 21716290 PMC3775282

[B48] Tervo D. G. Hwang B. Y. Viswanathan S. Gaj T. Lavzin M. Ritola K. D.. (2016). A designer AAV variant permits efficient retrograde access to projection neurons. Neuron 92, 372–382. 10.1016/j.neuron.2016.09.021 27720486 PMC5872824

[B49] Trouche S. Koren V. Doig N. M. Ellender T. J. El-Gaby M. Lopes-Dos-Santos V.. (2019). A hippocampus-accumbens tripartite neuronal motif guides appetitive memory in space. Cell 176, 1393.e16–1406.e16. 10.1016/j.cell.2018.12.037 30773318 PMC6424821

[B50] Wiegert J. S. Mahn M. Prigge M. Printz Y. Yizhar O. (2017). Silencing neurons: tools, applications and experimental constraints. Neuron 95, 504–529. 10.1016/j.neuron.2017.06.050 28772120 PMC5830081

[B51] Yamamoto J. Tonegawa S. (2017). Direct medial entorhinal cortex input to hippocampal CA1 is crucial for extended quiet awake replay. Neuron 96, 217.e4–227.e4. 10.1016/j.neuron.2017.09.017 28957670 PMC5672552

[B52] Zhu Y. Wienecke C. F. Nachtrab G. Chen X. (2016). A thalamic input to the nucleus accumbens mediates opiate dependence. Nature 530, 219–222. 10.1038/nature16954 26840481 PMC4814115

