# Supplementary figures and images for: Off-Target Influences of Arch-Mediated Axon Terminal Inhibition on Network Activity and Behavior
Source: Front Neural Circuits. 2020 Mar 25;14:10. doi: 10.3389/fncir.2020.00010 (PMC7109268; doi:10.3389/fncir.2020.00010)

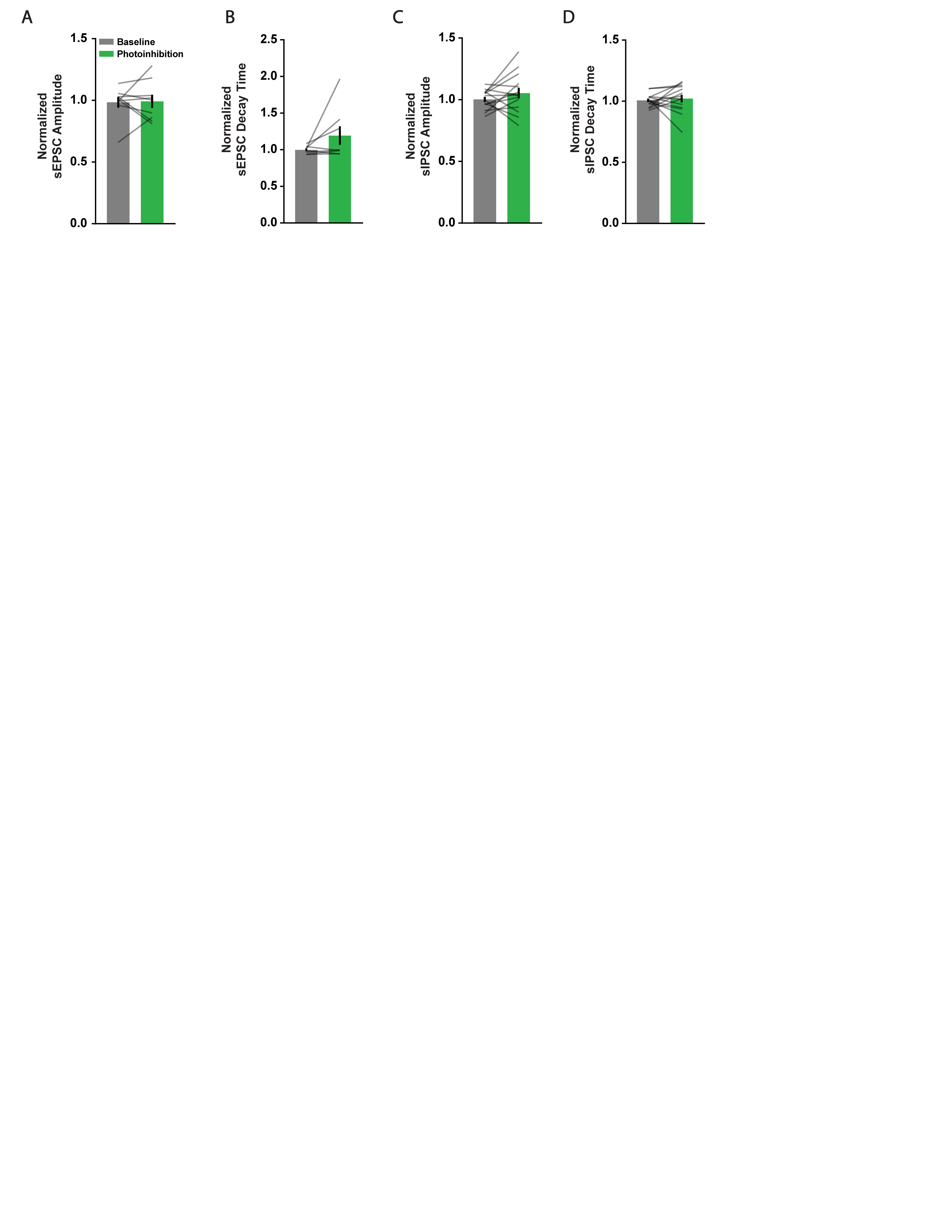

Supplement: FIGURE S1 — ArchT-mediated photoinhibition of excitatory axons in the NAc does not affect the amplitude or the decay rate of spontaneous synaptic currents. Related to Figures 2, 3. Summary of effect of ArchT axon terminal inhibition on (A) the amplitude of spontaneous EPSCs [n = 9(3 animals); t(8) = 0.13, p = 0.90], (B) the decay rate of spontaneous EPSCs (n = 8(3); t(7) = 1.61, p = 0.15], (C) the amplitude of spontaneous IPSCs [n = 14(6); t(13) = 1.22, p = 0.25], and (D) the decay rate of spontaneous IPSCs [n = 14(6); t(13) = 0.46, p = 0.65]. [file Image_1.tif]

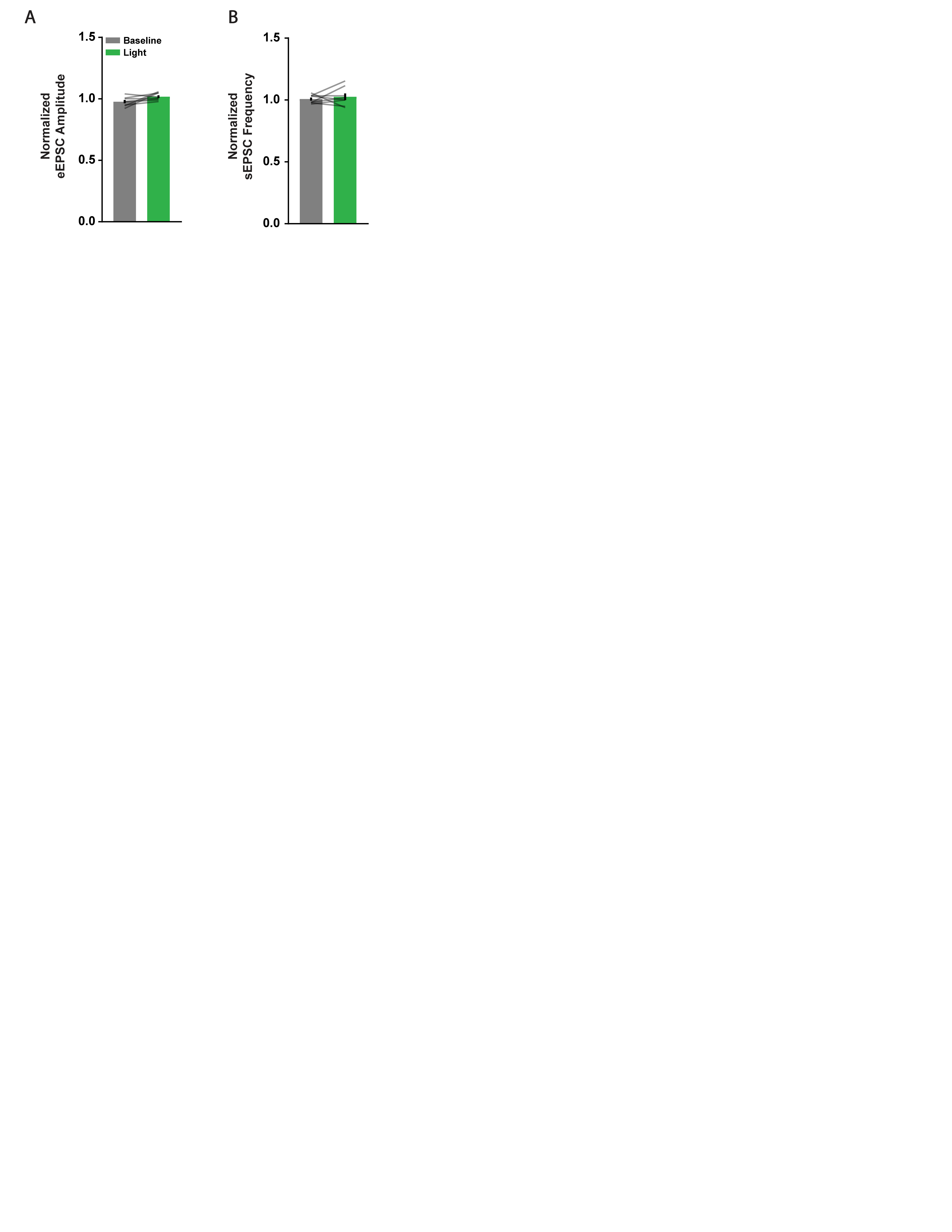

Supplement: FIGURE S2 — Light alone does not affect the amplitude of eEPSCs or the frequency of sEPSCs in NAc cell recordings from wildtype animals. Related to Figure 2. Summary of the effect of light on (A) the amplitude of evoked EPSCs [n = 8(3 animals); t(7) = 2.11, p = 0.07] and (B) the frequency of spontaneous EPSCs (t(7) = 0.63, p = 0.55). [file Image_2.tif]

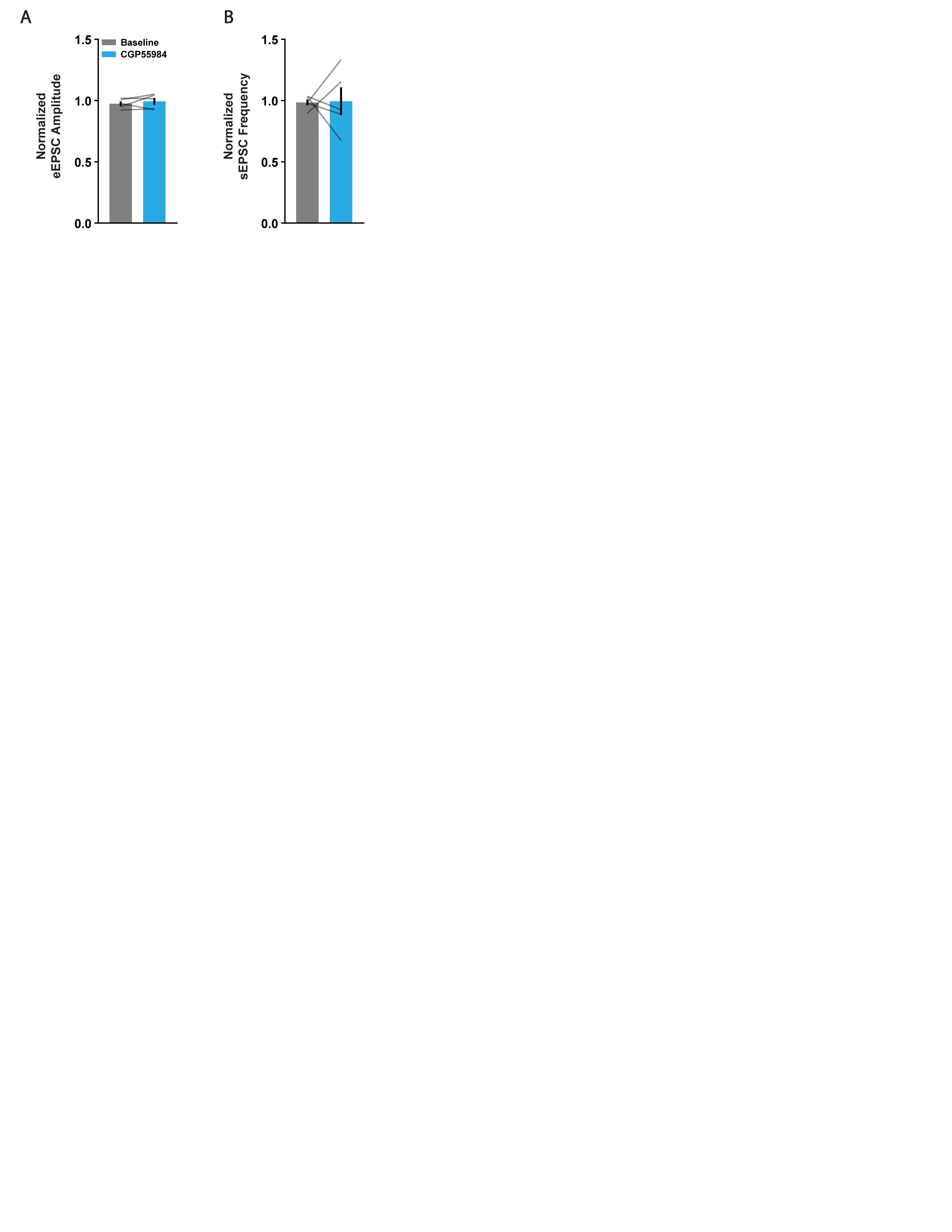

Supplement: FIGURE S3 — In the absence of photoinhibition, a GABAB antagonist does not affect the amplitude of eEPSCs or the frequency of sEPSCs recorded from NAc neurons of wildtype animals. Related to Figure 3. Summary of the effect of light on (A) the amplitude of evoked EPSCs [n = 5(3 animals); t(4) = 0.80, p = 0.47] and (B) the frequency of spontaneous EPSCs (t(4) = 0.08, p = 0.94). [file Image_3.tif]
